# Pathological Impact of Hepatitis B Virus Surface Proteins on the Liver Is Associated with the Host Genetic Background

**DOI:** 10.1371/journal.pone.0090608

**Published:** 2014-03-04

**Authors:** Yuri Churin, Martin Roderfeld, Johannes Stiefel, Tilman Würger, Dirk Schröder, Tomomitsu Matono, Hans-Joachim Mollenkopf, Roberta Montalbano, Malvika Pompaiah, Kurt Reifenberg, Daniel Zahner, Matthias Ocker, Wolfram Gerlich, Dieter Glebe, Elke Roeb

**Affiliations:** 1 Department of Gastroenterology, Justus Liebig University, Giessen, Germany; 2 Core Facility Microarray, Max Planck Institute for Infection Biology, Berlin, Germany; 3 Institute for Surgical Research, Philipps University of Marburg, Marburg, Germany; 4 Department of Molecular Biology, Max Planck Institute for Infection Biology, Berlin, Germany; 5 Central Laboratory Animal Facility, Johannes Gutenberg University, Mainz, Germany; 6 Division of Animal Welfare and Ethology, Justus Liebig University, Giessen, Germany; 7 Institute of Medical Virology, National Reference Centre for Hepatitis B and D Viruses, Justus Liebig University, Giessen, Germany; Singapore Institute for Clinical Sciences, Singapore

## Abstract

**Background:**

While the immune pathogenesis caused by hepatitis B virus (HBV) infection has been studied extensively, little is known about direct pathogenic effects of HBV surface proteins. Here, we have investigated pathological cellular effects of HBV surface protein expression in the liver of transgenic mice with different genetic background.

**Methods:**

The impact of HBV surface protein expression on the liver was studied in two mouse strains, BALB/c and C57BL/6. Histology and hydroxyproline assays were performed to investigate liver morphology and fibrosis. Gene expression and signaling were analyzed by microarray, qPCR and Western blotting.

**Results:**

Expression of HBV surface proteins in the liver of transgenic mice induced activation of protein kinase-like endoplasmic reticulum kinase (PERK) and eukaryotic initiation factor 2α (eIF2α) phosphorylation. Phosphorylation of eIF2α resulted in activation of the ER stress markers glucose regulated protein (GRP) 78 and pro-apoptotic C/EBP homologous protein (CHOP) in transgenic mice on BALB/c genetic background leading to stronger liver injury and fibrosis in comparison with transgenic mice on C57BL/6 background. Hepatic stellate cells represented the main collagen-producing liver cells in HBV transgenic mice. The key regulators of hepatocyte proliferation, transcription factors c-Jun and STAT3 were activated in HBV transgenic mice. Tumour incidence in transgenic mice was strain- and sex-dependent.

**Conclusions:**

Extent of liver injury, fibrosis, and tumour development induced by hepatic HBV surface protein expression considerably depends on host genetic background.

## Introduction

Chronic infection with hepatitis B virus (HBV) affects 350 to 400 million individuals worldwide and is the leading cause of liver cirrhosis and hepatocellular carcinoma worldwide [1]. Although much is known about HBV structure and replicative cycle [2] the pathogenic mechanisms responsible for liver injury, cirrhosis development and malignant transformation during chronic HBV infection are not well understood. It is believed that these events originate from persistent immune pathogenesis [3], but observations in transgenic mouse models of HBV infection suggest that in absence of an adaptive immune responses cellular mechanisms induced by HBV proteins may also lead to the development of these liver diseases [4], [5].

The HBV transgenic mice used in the current study contain a sub-genomic HBV DNA fragment (genotype D, subtype *ayw*) encoding the three co-carboxyterminal HBV surface (HBs) proteins under the control of the liver-specific murine albumin promoter [6]. Although these transgenic mice have been shown to develop chronic liver injury, regenerative hyperplasia, as well as adenomas and hepatocellular carcinomas (HCC) [7], [8] the mechanisms of HBs proteins pathogenicity are poorly understood. The HBs protein expression pattern in this mouse model mimics the situation in the liver of patients with enhanced intracellular expression and retention of Hepatitis B virus surface proteins, e. g. patients with Hepatitis B virus-related chronic liver disease treated by transplantation. The liver damage in these patients was attributable to a direct hepatocytotoxic effect of HBV, since they were on a similar immunosuppresion regime [9], [10].

Accumulation of misfolded proteins in the ER activates the unfolded protein response (UPR) that is sensed by the binding immunoglobulin protein (BiP)/glucose-regulated protein 78 (GRP78) [11]–[13]. Distinct branches of UPR are mediated by three different classes of ER-membrane transducers: inositol-requiring protein-1 (IRE1), activating transcription factor-6 (ATF6) or protein kinase-like endoplasmic reticulum kinase (PERK). PERK activation causes the phosphorylation of the alpha subunit of eukaryotic translation-initiation factor 2 (eIF2α) [11]. Phosphorylation of eIF2α leads to a reduction in the initiation of mRNA translation thus reducing the load of new proteins that require folding in the ER. However, the expression of some proteins is enhanced. One of them is the C/EBP homologous protein (CHOP), also known as growth arrest and DNA damage-inducible gene (GADD) 153 that mediates pro-apoptotic pathways emanating from the stressed ER [11], [14]. Previously it was shown that GRP78 expression was increased in a human hepatoma cell line that overproduced HBs proteins [15] and in the liver of transgenic mice that expressed deletion mutant of large HBs [16].

Hepatic fibrosis constitutes the wound healing response to liver injury. During fibrosis, hepatic stellate cell (HSC) activation represents a critical event, because these cells become the primary source of extracellular matrix in the liver upon damage [17]. Development of hepatic fibrosis after chemical liver injury is enhanced in BALB/c mice exhibiting a Th2 response compared to C57BL/6 mice, which demonstrated a primary Th1 response [18]. Transgenic mice on fibrosis-resistant C57BL/6 genetic background, which over-produce HBs proteins, develop modest spontaneous liver fibrosis [19], [20].

Transcription factor c-Jun and signal transducer and activator of transcription (STAT) 3 are implicated in several cellular processes including proliferation, survival, and cell transformation [21], [22]. They are activated in chemically induced murine liver tumours and in HCCs of humans [23], [24], suggesting an important function for these proteins in the development of liver tumours.

Here we report that the production of HBV surface proteins stimulates the expression of CHOP in hepatocytes and could cause stronger liver damage in transgenic mice on BALB/c genetic background compared to C57BL/6. Furthermore, HBV transgenic mice develop hepatic fibrosis and the level of fibrosis depends on the genetic background. Although c-Jun transcription factor up-regulation and activation of STAT3 and PERK in the liver of transgenic mice might contribute to tumour development, CHOP expression might reduce tumorigenesis in transgenic mice on BALB/c genetic background.

## Materials and Methods

### Animal Model

Transgenic mice were maintained at the Central Animal Laboratory of the Justus-Liebig-University Giessen under specified pathogen-free conditions. This study was carried out in strict accordance with the recommendations in the Guide for the Care and Use of Laboratory Animals of the German law of animal welfare. The mice received humane care, and all experiments were approved by the Committee on the ethics of Animal Experiments of the Regierungspraesidium Giessen, Giessen, Germany (permit number: V54-19c 20 15 h 01 GI20/10 Nr. 52/2011 and Nr. A5/2012).

Generation and characteristics of transgenic lineages Tg(Alb-1HBV), internal designation (HBVTg/6) have been described previously [8]. The HBVTg/6 strain had an inbred C57BL/6 genetic background and was propagated by crossing hemizygous transgenic males to C57BL/6 females. These mice were backcrossed to fibrosis susceptible BALB/c genetic background [18] for at least 6 generations. The obtained transgenic mouse line was internally designed HBVTg/c. At age of 12, 26, and 52 weeks mice were killed by CO_2_-inhalation. Liver samples were collected and preserved for analyses in accordance with application. Serum samples were stored at −80°C until analysis of alanine aminotransferase (ALT) by routine clinical chemistry on a Reflotron Plus Analyzer (Roche, Mannheim, Germany).

### Histology and Hydroxyproline Assay

Immediately after necropsy, liver samples for histology were fixed in 4% neutral buffered paraformaldehyde at 4°C for 16 hours and embedded in paraffin. Paraffin sections (5 µm) were stained with hematoxylin and eosin (H&E) or 0.1% Sirius Red F3B in saturated picric acid (Chroma, Münster, Germany) for the detection of collagen fibers [25]. The entire content of collagen was determined by hydroxyproline (HYP) quantification [26].

### Immunohistochemistry

Immunohistochemistry (IHC) was performed using ImmPRESS Peroxidase Detection Reagents (Vector Laboratories) and antibodies specific for HBsAg (20-HR20), GADD153 (F-168, Santa Cruz), Desmin and GFAP (Lab Vision), c-Jun (60A8, Cell Signaling), BiP (C50B12, Cell Signaling). Colour reaction was developed with VECTOR VIP Peroxidase Substrate Kit or DAB Peroxidase Substrate Kit, (Vector Laboratories). The percentage of BiP, Desmin, and GFAP-positive area was determined using ImageJ image analysis system (National Institutes of Health, Bethesda, MD, USA).

### Western Blot Analysis

Total liver lysates were analyzed by immunoblotting using antibodies against HBsAg (20-HR20, Fitzgerald), GADD153 (F-168, Santa Cruz), phospho-PERK (16F8, Cell Signaling), PERK (H-300, Santa Cruz), phospho-eIF2α (119A11, Cell Signaling), eIF2α (L57A5, Cell Signaling), β-actin (13E5, Cell Signaling), JNK2 (N-18, Santa Cruz), c-Jun (60A8, Cell Signaling), phospho-c-Jun (D47G9, Cell Signaling), phospho-SAPK/JNK (81E11, Cell Signaling), STAT3 (79D7, Cell Signaling), phospho-STAT3 (D3A7, Cell Signaling).

### Assay for HBV-specific proteins

HBsAg was measured in serum and in liver lysates by an in-house sandwich ELISA as described [27]. HBsAg amount per hepatocyte was calculated based on the hepatocellularity number for mouse 135 million cells per gram of liver [28].

### Quantitative real-time PCR (qPCR)

RNA isolation, cDNA synthesis, qPCR and quality control of all steps were performed as described previously [29]. Primers were purchased from QIAGEN (Hilden, Germany). qPCR data were analysed using ΔΔCt method [30].

### Microarray analysis

Microarray experiments were performed with total RNA from the liver of 12-week-old mice as described previously [31]. The data presented here have been deposited in NCBI's Gene Expression Omnibus (GEO; http://www.ncbi.nlm.nih.gov/geo/) and are accessible through GEO Series accession number GSE40826.

### Statistical analysis

Statistical analysis was performed with SPSS V.17.0 software (SPSS Inc.). For non-normally distributed parameters, Mann-Whitney U test and Spearman rank test were applied. The results are presented as mean±SEM. A two-sided p<0.05 was considered significant (shown as * in the figures].

## Results

### Liver damage induced by HBs proteins expression depends on host genetic background

**Figure 1:**
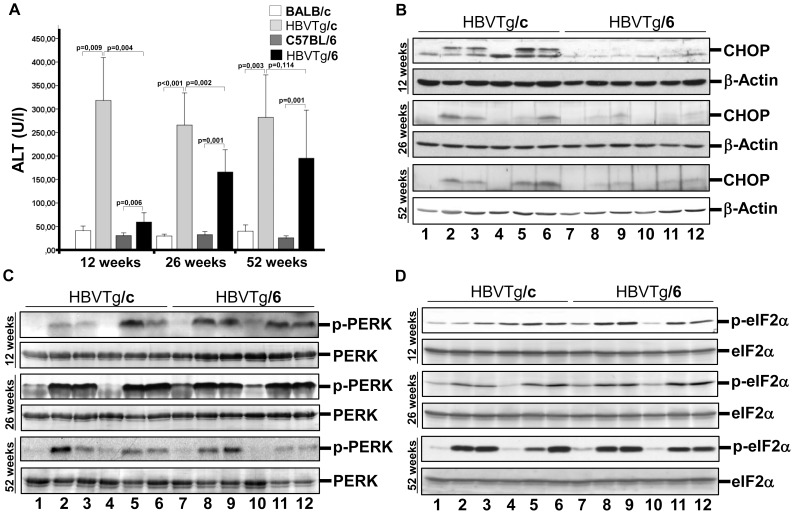
HBs proteins-induced liver injury depends on host genetic background. (A) Assessment of liver damage in wild-type and HBV transgenic mice. ALT was measured in serum of 12-, 26-, and 52-week-old mice (mean±SEM, n = 5–10). (B-D) Western blot analysis of total protein lysates from the liver of 12-, 26-, and 52-week-old mice was performed using (B) anti-CHOP/GADD153, (C) anti-phospho-PERK, and (D) anti-phospho-eIF2α (Ser51) antibodies. Equal protein loading was confirmed with anti-PERK (B), anti-eIF2α (C), and anti-β-actin (D) antibodies. **1** – female BALB/c; **2,3** – female HBVTg/c; **4** – male BALB/c; **5, 6** – male HBVTg/c; **7** – female C57BL/6; **8, 9** – female HBVTg/6; **10** – male C57BL/6; **11, 12** – male HBVTg/6 mice.

Transgenic mice were sacrificed at week 12, 26, and 52. To estimate the level of liver cell damage we have measured alanine transaminase (ALT) in serum. Activities of serum ALT were markedly increased already in 12-week-old HBVTg/c mice compared to the wild-type and remained at that level during the observation period. In HBVTg/6 mice the ALT elevation was moderate at week 12 but increased later to levels almost as high as in HBVTg/c mice (Figure 1A). Histological analysis revealed no significant lymphocyte infiltration of the liver from 12-week-old transgenic mice (Figure S1A). Furthermore, amounts of HBsAg per hepatocyte in 12-week-old mice were similar: 0.42 pg/cell (range, 0.18–0.71 pg/cell; n = 5) for HBVTg/c and 0.31 pg/cell (range, 0.15–0.64 pg/cell; n = 5) for HBVTg/6. Immunohistochemical and Western blot analyses of livers from transgenic mice also showed that distributions and expression levels of HBs proteins were similar in mice on both C57BL/6 and BALB/c genetic backgrounds (Figure S1B and S2). Moreover, amounts of HBsAg in serum of transgenic mice did not differ significantly: 2.02 ng/ml (range, 0.68–3.19 ng/ml; n = 5) for HBVTg/c and 1.34 ng/ml (range, 0.68–2.55 ng/ml; n = 4) for HBVTg/6. Thus, the elevated serum ALT levels in HBVTg/c mice could be a consequence of a specific host response to the presence of HBs proteins.

To identify the pathways which are activated in the HBs-expressing liver tissue we carried out transcriptional profiling using microarray analysis. As illustrated in Table 1 a set of genes that is known to be induced by UPR is up-regulated in the liver of 12-week-old transgenic mice. Interestingly, the expression of gene *ddit3* that encodes CHOP protein is stronger up-regulated in the liver of HBVTg/c mice compared to HBVTg/6. To confirm this finding we studied the expression of CHOP by qRT-PCR and Western blot analysis. Transcription of the *ddit3* gene was strongly activated in 12-week-old HBVTg/c mice and declined thereafter with age, whereas in HBVTg/6 mice *ddit3* transcription was rather weakly enhanced in 12-week-old mice and increased with age (Figure S3). Western blot analysis of liver proteins revealed that CHOP was up-regulated much stronger in HBVTg/c mice compared to HBVTg/6 (Fig. 1B). Selective CHOP translation depends on eIF2α phosphorylation by PERK after induction of UPR [11]. However, we detected similar level of PERK activation and eIF2α phosphorylation in the liver from both mouse strains irrespective of gender and age (Fig. 1C and D). Interestingly, other UPR sensors ATF6 and IRE1 as well as eIF2α kinases PKR (protein kinase R) and GCN2 (general control non-derepressible-2) [32] were not activated in the liver of transgenic mice (data not shown). Taken together, expression of HBs proteins in the liver of transgenic mice leads to the specific activation of the PERK branch of UPR in hepatocytes from both mouse strains, but expression of CHOP was much stronger in HBVTg/c mice. Enhanced translation of CHOP results in liver damage and could explain higher serum ALT levels in HBVTg/c mice.

**Table 1:** Selected hepatic genes significantly differentially expressed in the liver of 12-week-old HBV transgenic mice.

Accession	Primary Sequence Name	Sequence Description	Fold Change HBVTg/c	Fold Change HBVTg/6
NM_019738	Nupr1	Nuclear protein 1	14.97	5.44
NM_007498	Atf3	Activating transcription factor 3	9.53	3.25
NM_007837	Ddit3	DNA-damage inducible transcript 3	6.39	2.14
NM_024440	Derl3	Der1-like domain family, member 3	8.52	1.44
NM_016773	Nucb2	Nucleobindin 2	4.16	1.81
NM_012055	Asns	Asparagine synthetase	4.14	2.28
NM_007836	Gadd45a	Growth arrest and DNA-damage-inducible 45 alpha	2.61	1.07
NM_144554	Trib3	Tribbles homolog 3	2.18	−1.13
NM_013560	Hspb1	Heat shock protein 1	2.14	2.01
NM_022310	Hspa5	Heat shock 70 kD protein 5 (GRP78)	2.08	1.19
NM_011631	Hsp90b1	Heat shock protein 90 kDa beta (Grp94)	1.91	−1.05
NM_011817	Gadd45g	Growth arrest and DNA-damage-inducible 45 gamma	−1.67	2.18
NM_010591	Jun	Jun oncogene	4.17	2.30
NM_008182	Gsta2	Glutathione S-transferase, alpha 2 (Yc2)	3.20	2.04
NM_031170	Krt2-8	Keratin complex 2, basic, gene 8	2.21	1.95
NM_007742	Col1a1	Procollagen, type I, alpha 1	2.00	1.48
NM_007743	Col1a2	Procollagen, type I, alpha 2	1.94	1.23
NM_011594	Timp2	Tissue inhibitor of metalloproteinase 2	1.75	−1.04
NM_010664	Krt1-18	Keratin complex 1, acidic, gene 18	1.81	1.80

**Figure 2:**
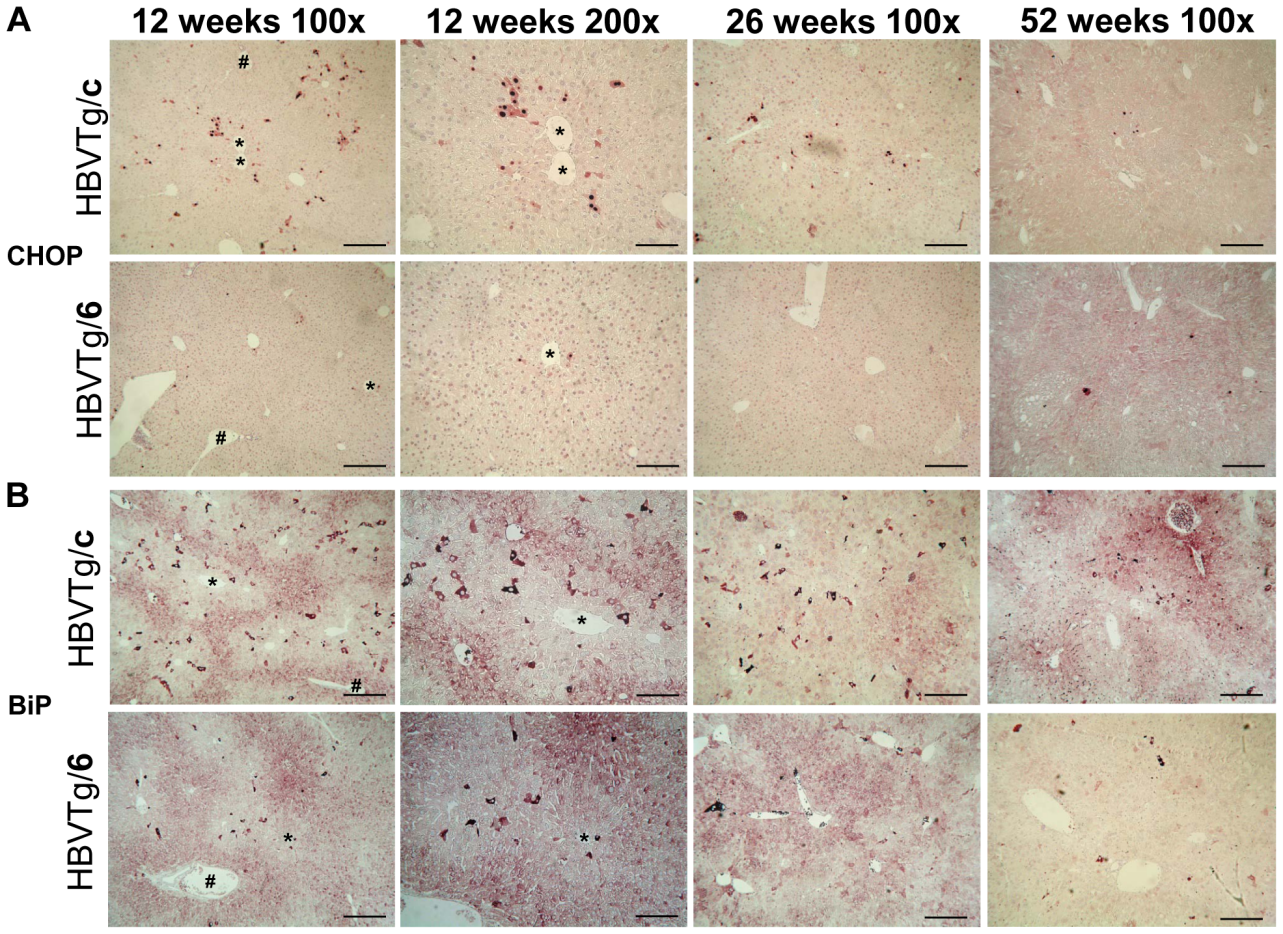
Expression of CHOP/GADD153 and BiP/GRP78 in the liver of HBV transgenic mice. Immunohistochemical analysis of paraffin-embedded liver sections from 12-, 26-, and 52-week-old mice was performed using specific anti-CHOP/GADD153 (A) and anti-BiP (B) antibodies. 100x-original magnification 100×, bar  = 200 µm. 200-original magnification 200×, bar = 100 µm. *-central vein. **#**-periportal vein.

To examine the location of CHOP expressing hepatocytes immunohistochemistry (IHC) was performed. According to our previous finding a significant part of hepatocytes from 12-week-old HBVTg/c mice accumulated CHOP in the nucleus and the amount of CHOP-positive hepatocytes declined with age, whereas we could detect only a few hepatocytes in HBVTg/6 liver positively stained for CHOP independent of age (Figure 2A). Interesting, CHOP-positive hepatocytes were located in centrilobular areas which surround a hepatic central vein (Figure 2A). Furthermore, induction of UPR leads to activation of the major sensor of unfolded protein accumulation BiP/GRP78 [12]. IHC demonstrated strong expression of BiP in selected hepatocytes in centrilobular areas (Figure 2B and Figure S4), although Western blot analysis did not reveal any differences between wild-type and transgenic mice in expression of BiP/GRP78 (data not shown). Thus, expression of HBs proteins activated the UPR downstream pathway (CHOP and BiP) much stronger in the liver of transgenic mice on BALB/c genetic background compared to C57BL/6. This activation is located in centrilobular zones of the liver.

### Liver fibrosis

**Figure 3:**
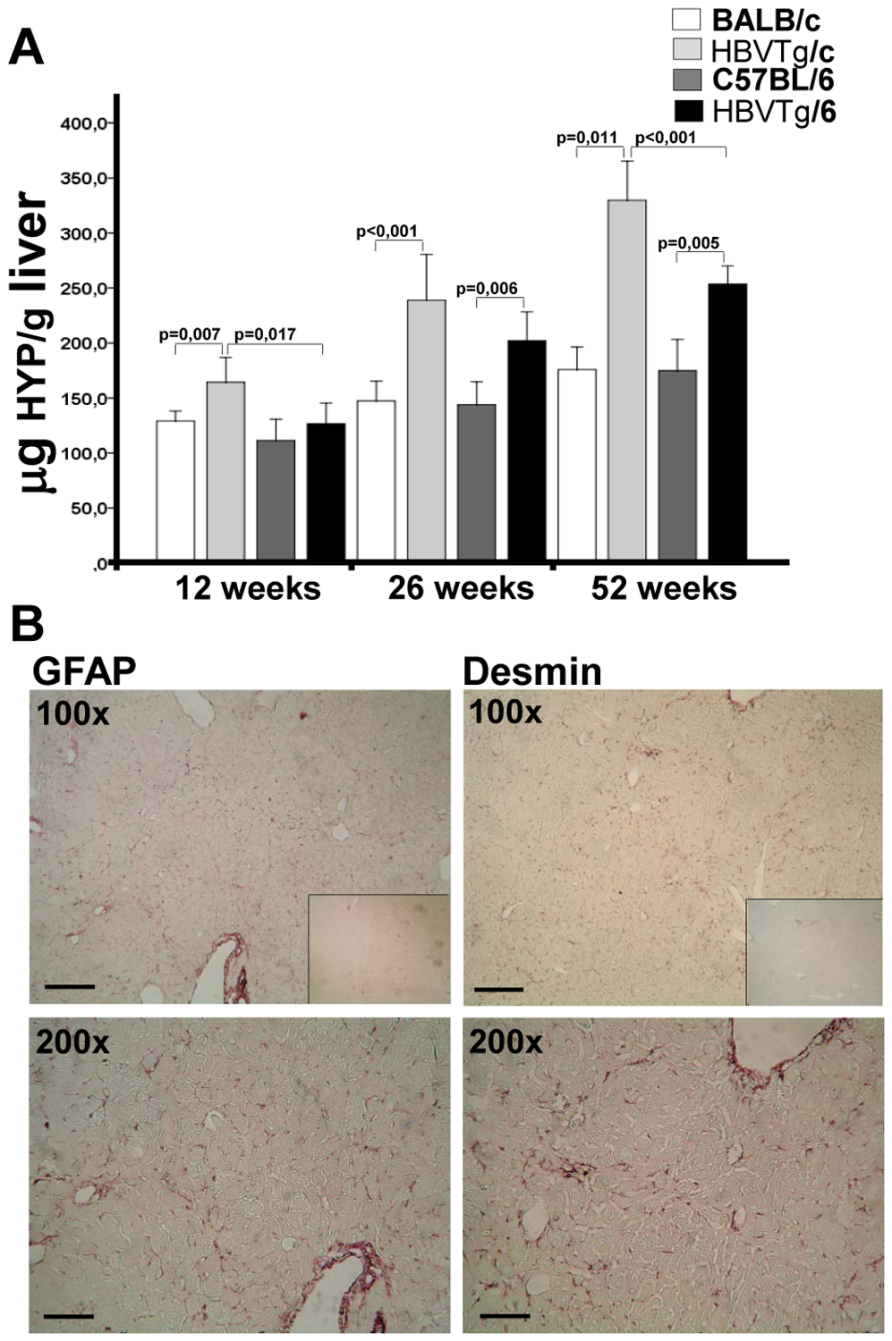
Fibrogenesis in the liver of HBV transgenic mice. (A) Entire collagen was assessed by measurement of hydroxyproline (HYP) in murine liver (mean±SEM, n = 5–10). (B) Activation of hepatic stellate cells in HBV transgenic mice. Paraffin-embedded liver sections of 26-week-old HBVTg/c mice were analysed using anti-GFAP (left panel) and anti-Desmin (right panel) antibodies. Inserts - immunohistochemical analysis of the liver from wild-type BALB/c mouse. 100× - original magnification 100×, bar  = 200 µm. 200× – original magnification 200×, bar  = 200 µm.

Measurement of liver hydroxyproline content demonstrated the development of hepatic fibrosis in transgenic mice (Figure 3A). Enhanced hepatic fibrosis was confirmed by Sirius red staining as well. We observed minimal fibrosis in the liver of 12-week-old mice. But fibrosis constantly increased with age. However, HBVTg/c mice accumulated more collagen (Figure S5). Hepatic stellate cells (HSC) are the primary effector cells responsible for the deposition of ECM in normal and fibrotic liver [33]. Therefore, we tested the expression of HSC activation markers. We detected increased amounts of GFAP- and desmin- positive cells in the liver of transgenic mice, thus demonstrating HSC proliferation in the liver of transgenic mice (Figure 3B and Figure S6). Moreover, double staining of desmin with specific antibodies and collagen with Sirius red has shown co-localization of HSCs with collagen fibres (Figure S7). Taken together, expression of HBs proteins in mouse liver induces development of hepatic fibrosis, which correlated with liver injury. HSCs might be the main collagen-producing cells in this mouse model.

### HBs protein-induced tumour development depends on host genetic background

**Figure 4:**
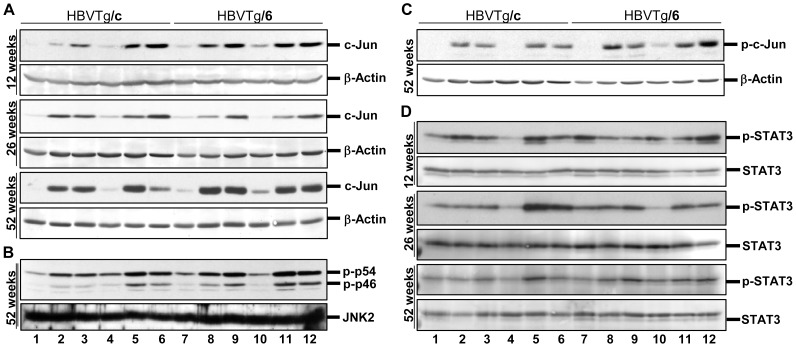
Activation of tumorigenic pathways in hepatocytes of HBV transgenic mice. Western blot analysis of total protein lysates from the liver of 12-, 26-, and 52-week-old mice was performed using (A) anti-Jun, (B) anti-phospho-SAPK/JNK, (C), anti-phospho-c-Jun and (D) anti-phospho-STAT3 antibodies. Samples were loaded as described in the legend of Fig. 1. Equal protein loading was confirmed with anti-β-actin (A, B and C) and anti-STAT3 (D) antibodies.

Microarray analysis showed an up-regulation of *c-jun* gene expression in transgenic mice independent of genetic background (Table 1). These results were confirmed using qPCR. Maximal expression was detected in the liver of 52-week-old mice (Figure S8). Expression of c-Jun protein was increased in the liver of 12-, 26-, and 52-week-old transgenic mice (Figure 4A). Major parts of hepatocytes of 52-week-old mice accumulated c-Jun in the nucleus (Figure S9). Phosphorylation of c-Jun by c-Jun N-terminal kinase (JNK) stimulates its ability to activate transcription [34], [35]. Western blot analyses demonstrated that JNKs were activated (Figure 4B) and the level of c-Jun phosphorylation was indeed increased in the liver of 52-week-old transgenic mice (Figure 4C). Thus, expression of HBs proteins in the liver of transgenic mice leads to activation of c-Jun expression.

STAT3 activation was observed in mouse models of liver injury and in human liver diseases in the context of inflammation and cancer [22]. Therefore, we examined the status of STAT3 activation in the liver of HBV transgenic mice. Western blot analysis of liver protein extracts revealed STAT3 activation in the liver of male but not female mice (Figure 4D). Thus, expression of HBV surface proteins in the liver of transgenic mice results in STAT3 activation in a gender-dependent manner.

Up-regulation of c-Jun expression and STAT3 activation could promote hepatic tumour growth [21], [22]. We checked transgenic mice for occurrence of liver tumour. In young mice (12- and 26-week-old) mice we could detect no tumours. However, they were detected in 100% (6/6) of 52-week-old male and 20% (1/5) of female HBVTg/6 mice, whereas only 58% (7/12; X^2^ = 3.46; p = 0.06) of 52-week-old male and 0% (0/6) of female HBVTg/c mice develop tumours. Hence, development of tumours in HBV transgenic mice was age-, gender-, and strain-dependent.

## Discussion

In this study we investigated the effects of HBVs proteins expression in the liver of transgenic mice BALB/c and C57BL/6 genetic background. Since we observed only weak strain-independent immune cell infiltration of transgenic mice liver (Figure S1) this model could be considered as one with impaired immune reaction. One important negative impact of immune deficiency on chronic HBV infection in human is related to the direct cytotoxicity of high levels of HBs and other HBV proteins [9], [10], [36]. Low serum HBsAG titers were associated with strong intracellular accumulation of HBs in HBV transgenic mice on both genetic backgrounds. This condition was also seen in some patients with late phases of chronic HBV infection [37], [38]. Thus, transgenic mice expressing HBs proteins reflect the situation in the liver of HBV-infected patients demonstrated strong retention of HBsAg in hepatocytes.

Higher serum ALT activities in HBVTg/c mice suggest stronger liver injury compared to HBVTg/6 (Figure 1A). Since the level of cellular infiltration was low in the liver of transgenic mice on both genetic backgrounds we searched for other reasons of hepatocyte death. Increased CHOP expression as a result of prolonged ER stress promotes cell death, whereas CHOP deletion protects against the death of ER-stressed cells [14]. Strongly increased transcription and protein accumulation of CHOP in HBVTg/c mice (Figure 1B and 2A; Figure S3) inducing hepatocyte death could explain increased serum ALT level in these mice. Expression of CHOP is mediated by phosphorylation of eIF2α [32] that in turn is phosphorylated by PERK [11]. Interestingly, levels of PERK activation and eIF2α phosphorylation were similar in the liver of both HBV transgenic mouse strains (Figure 1C and 1D). Two other branches of UPR IRE1α and ATF6 were not activated in the liver of HBV transgenic mice (data not shown). PERK branch activation is largely sustained with unmitigated ER stress, whereas persistent ER stress attenuates IRE1α and ATF6 signaling [39]. Therefore, permanent expression of HBs proteins leads to the activation of persistent ER stress in hepatocytes that induces PERK and impairs another branches of UPR. It is possible that this situation is common for chronic liver disease comprising ER stress induction.

It was previously shown that despite PERK activation and eIF2α phosphorylation in the liver of patients with nonalcoholic fatty liver disease (NAFLD) and nonalcoholic steatohepatitis (NASH), downstream effectors such as CHOP remain inactive [40]. A similar situation was observed in the liver of HBV transgenic mice on C57BL/6 genetic background. However, stimulation of CHOP and BiP expression in HBVTg/c mice demonstrated that the outcome of UPR induction depends on the genetic background of subjects. Furthermore, several studies have demonstrated that PERK function is critical for maintaining cellular redox homeostasis, promotes cancer cell proliferation and tumour growth [41]. Thus, sustained activation of PERK could also promote cancer development in the liver of HBV transgenic mice.

Global reduction of translation initiation due to PERK-mediated eIF2α phosphorylation [32] should also affect the expression of HBs proteins in the liver. This suggests the following feed-back mechanism: PERK activation results in the reduction of HBs translation and that leads to a balance between PERK activation and HBs protein synthesis in hepatocytes.

Development of tumours in HBV transgenic mice as it was shown by us and others [8] is age-, gender-, and strain-dependent. In this study we observed a strong up-regulation of c-Jun hepatic expression and an activation of STAT3, whose role in tumorigenesis is well established [22], [23], [42]. Jun controls liver cancer initiation and is required for development of chemically induced HCC [23]. Interestingly, transgenic mice comprising the whole or partial HBV genome are also more susceptible to chemically induced hepatocarcinogenesis [43], [44]. Likewise, hepatitis C virus core protein potentiates chemically induced HCC through c-Jun and STAT3 activation [45]. Thus, stimulation of c-Jun expression and STAT3 activation by HBs proteins could promote the development of liver cancer induced by different causes, such as sustained inflammation, activation of oncogenes etc. Furthermore, the finding that STAT3 was activated in male mice only correlated with our observation that tumour development in HBV transgenic mice is gender-dependent. There is accumulating evidence that tumour-specific ER stress can be exploited for cancer therapy by treatment with ER stress-aggravating compounds [46]. Moderate, transient ER stress response represents an adaptive mechanism to support cellular survival. However, severe and excessive stress conditions could turn this response system to its pro-apoptotic mode. Stimulation of CHOP expression in HBVTg/c mice indicated an activation of pro-apoptotic cellular stress responses in the liver and resulted in reduced tumour incidence in 52-week-old HBVTg/c mice.

Taken together, the outcome of HBV surface proteins expression in the liver of transgenic mice depends on the host genetic background. Liver injury and fibrosis were increased in transgenic mice on BALB/c background compared to C57BL/6 correlating with strong expression of PERK downstream pro-apoptotic effector CHOP. More interesting finding is genetic background-independent stimulation of c-Jun expression together with STAT3 and PERK activation promoting cancer cell proliferation and tumour growth. However, activation of pro-apoptotic cellular stress response could result in reduced tumour incidence in the liver.

## Supporting Information

Figure S1 **Representative liver histology and distribution of HBs proteins in hepatocytes of transgenic mice.** (A) H&E staining of liver sections from 12-, 26-, and 52-week-old mice HBVTg/c and HBVTg/6 mice. Original magnification 100×, bar  = 200 µm. Insets – H&E staining of liver from corresponding wild-type mice. (B) Paraffin-embedded sections of transgenic mice liver were stained with an antibody against HBsAg. Original magnification 100×, bar  = 200 µm.
(TIF)

Figure S2 **Expression of HBs proteins in the liver of transgenic mice.** Western blot analysis of total protein lysates from livers of HBV transgenic mice using specific anti-HBsAg antibody. **1**- female BALB/c; **2,3**- female HBVTg/c; **4** – male BALB/c; **5, 6** – male HBVTg/c; **7** – female C57BL/6; **8, 9** – female HBVTg/6; **10** – male C57BL/6; **11, 12** – male HBVTg/6 mice. Equal protein loading was confirmed with anti-β-actin antibody.
(TIF)

Figure S3 **Transcriptional up-regulation of** ***ddit3*** **gene in the liver of HBV transgenic mice.** All data are normalized to r18S. Fold increase to wild-type animals is depicted (mean ± SEM, n = 5–10).
(TIF)

Figure S4 **Quantification of BiP expression in the liver of HBV transgenic mice.** Percentage of BiP-positive area in the whole image was estimated with ImageJ software (mean±SEM, n = 5).
(TIF)

Figure S5 **Development of fibrosis in the liver of HBV transgenic mice.** Collagen fibres are detected in polarized light after Sirius red staining of 5-µm paraffin-embedded liver sections of 12-, 26-, and 52-week-old HBVTg/c and HBVTg/6 mice. Original magnification 100×, bar  = 200 µm. Insets – Sirius red staining of the liver from wild-type mice.
(TIF)

Figure S6 **Quantification of Desmin and GFAP expression in the liver of HBV transgenic mice.** Percentages of Desmin (A) and GFAP (B)-positive areas in the whole images were estimated with ImageJ software (mean±SEM, n = 5).
(TIF)

Figure S7 **Hepatic stellate cells are major contributor to hepatic fibrosis in HBV transgenic mice.** Paraffin-embedded liver section from 26-week-old HBVTg/c mouse was first stained with anti-Desmin antibody and subsequently with Sirius red. Collagen fibres are detected in polarized light, Desmin-positive staining appears in black. Original magnification 200×, bar  = 100 µm.
(TIF)

Figure S8 **Transcriptional up-regulation of** ***c-jun*** **gene in the liver of HBV transgenic mice.** All data are normalized to r18S. Fold increase to wild-type animals is depicted (mean ± SEM, n = 5–10).
(TIF)

Figure S9 **Expression of Jun protein in the liver of HBV transgenic mice.** Immunohistochemical analysis of paraffin-embedded liver sections from 52-week-old mice was performed using specific anti-Jun antibody. Original magnification 100×, bar  = 200 µm.
(TIF)
