# Correction: Pathological Impact of Hepatitis B Virus Surface Proteins on the Liver Is Associated with the Host Genetic Background

**DOI:** 10.1371/journal.pone.0127375

**Published:** 2015-05-01

**Authors:** Yuri Churin, Martin Roderfeld, Johannes Stiefel, Tilman Würger, Dirk Schröder, Tomomitsu Matono, Hans-Joachim Mollenkopf, Roberta Montalbano, Malvika Pompaiah, Kurt Reifenberg, Daniel Zahner, Matthias Ocker, Wolfram Gerlich, Dieter Glebe, Elke Roeb

In Fig 4A of the original published article, some of the bands from the c-Jun at 26 weeks panel, were run on a single gel, but were not adjacent in the original gel and were spliced together to improve ease of readability. In the revised image the authors have provided the unspliced panel indicating that for this panel Lane 9 is male C57BL/6 and Lane 10 is female HBVTg/6 unlike in the other panels where Lane 9 is female HBVTg/6 and Lane 10 is male C57BL/6. In addition, the authors reproduced the beta-actin control for both Week 26 and 52. In the revised figure, the authors have provided the correct beta-actin control for Week 52. The original raw files for the gel images are provided as supplementary files in addition to the revised figure. The authors apologize for the errors and any inconvenience that it may have caused.

**Fig 4:**
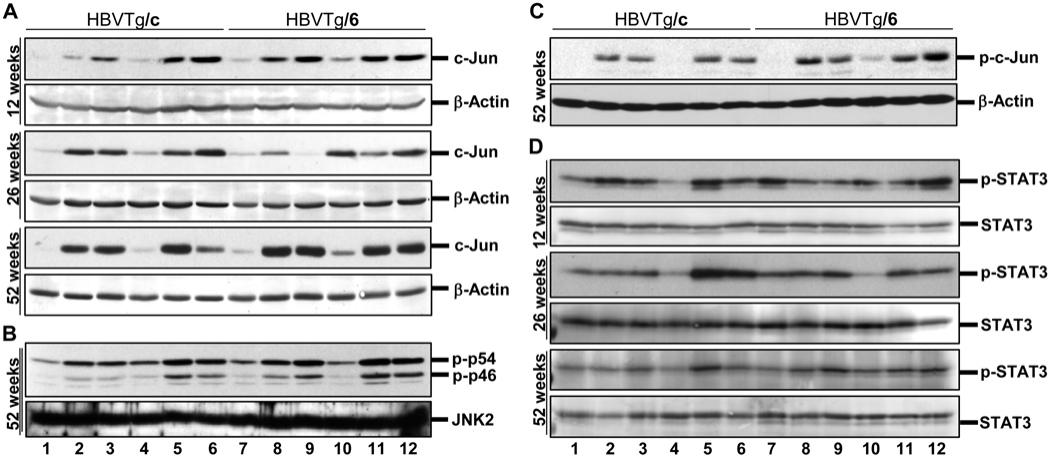
Activation of tumorigenic pathways in hepatocytes of HBV transgenic mice. Western blot analysis of total protein lysates from the liver of 12-, 26-, and 52-week-old mice was performed using (A) anti-Jun, (B) anti-phospho-SAPK/JNK, (C), anti-phospho-c-Jun and (D) anti-phospho-STAT3 antibodies. Samples were loaded as described in the legend of Fig 1 except (A) panel 26 weeks, where the loading was as follows: **1**—female BALB/c; **2,3**—female HBVTg/c; **4**—male BALB/c; **5, 6**—male HBVTg/c; **7**—female C57BL/6; **8**—female HBVTg/6; **9**—male C57BL/6; **10**—female HBVTg/6, **11, 12**—male HBVTg/6 mice. Equal protein loading was confirmed with anti-β-actin (A, B and C) and anti-STAT3 (D) antibodies.

## Supporting Information

S1 File c-Jun 26 Weeks (TIF)

S2 File Actin 26 Weeks (TIF)

S3 File Actin 52 Weeks (TIF)
