# Supplementary figures and images for: Pathological Impact of Hepatitis B Virus Surface Proteins on the Liver Is Associated with the Host Genetic Background
Source: PLoS One. 2014 Mar 4;9(3):e90608. doi: 10.1371/journal.pone.0090608 (PMC3942466; doi:10.1371/journal.pone.0090608)

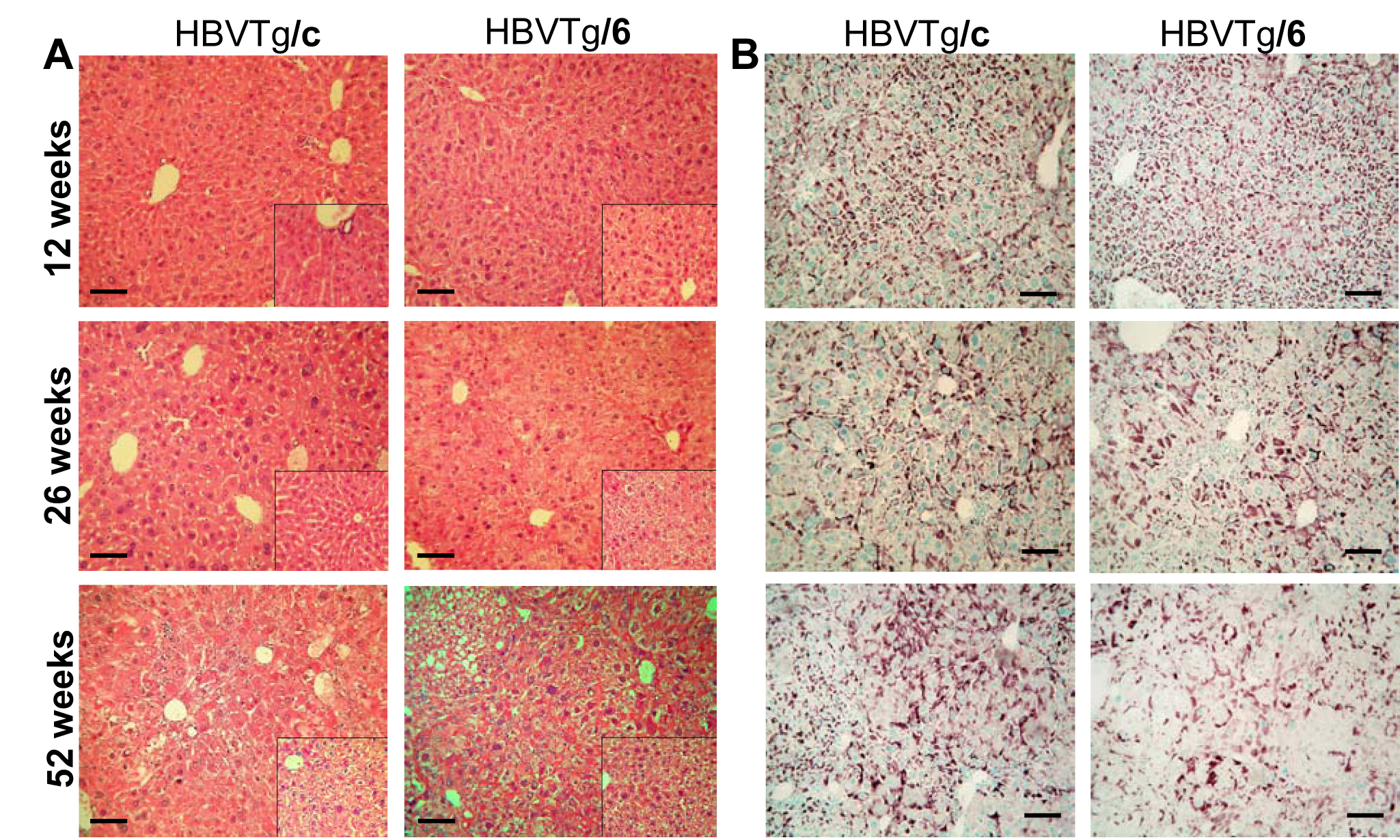

Supplement: Figure S1 — Representative liver histology and distribution of HBs proteins in hepatocytes of transgenic mice. (A) H&E staining of liver sections from 12-, 26-, and 52-week-old mice HBVTg/c and HBVTg/6 mice. Original magnification 100×, bar = 200 µm. Insets – H&E staining of liver from corresponding wild-type mice. (B) Paraffin-embedded sections of transgenic mice liver were stained with an antibody against HBsAg. Original magnification 100×, bar = 200 µm. (TIF) [file pone.0090608.s001.tif]

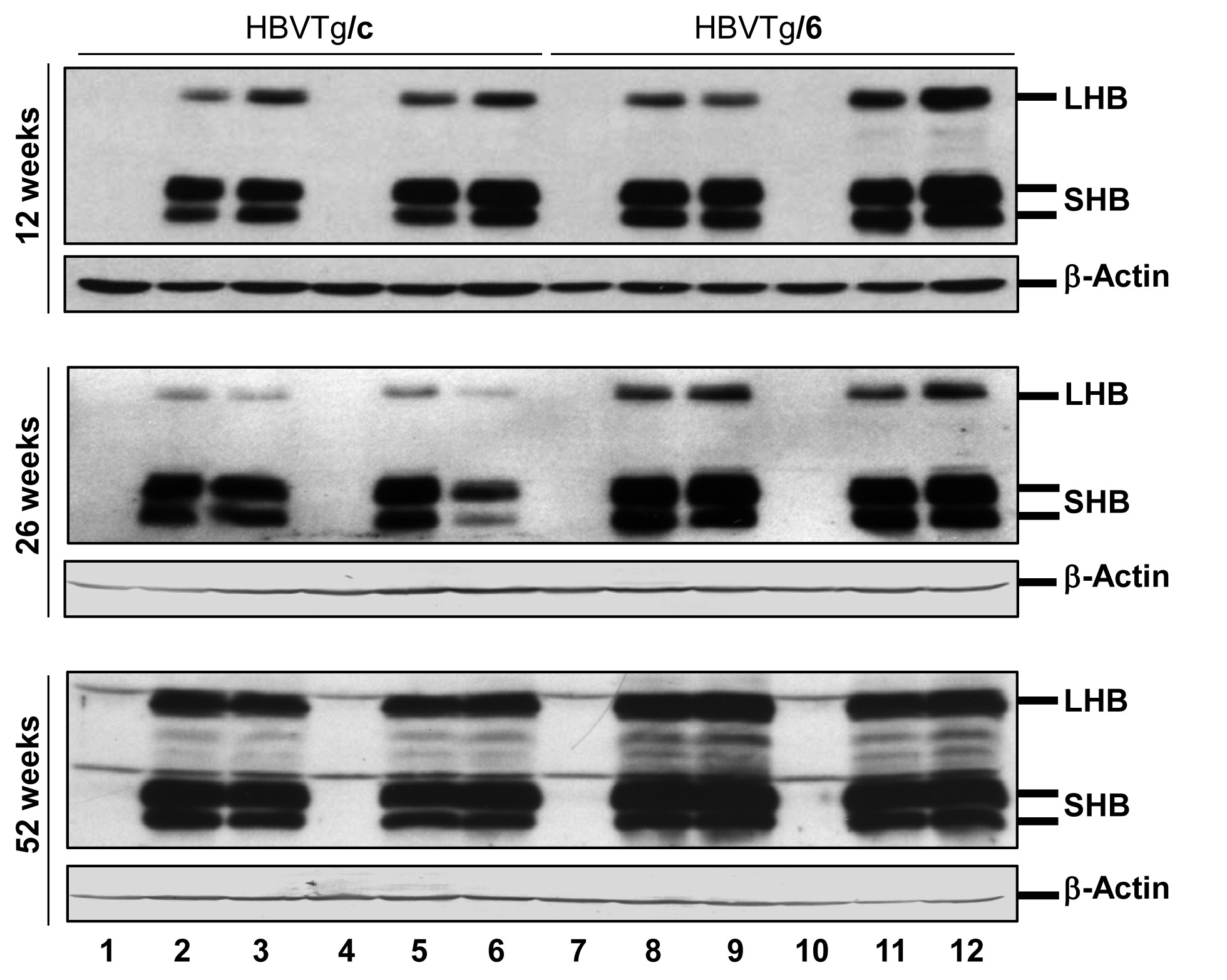

Supplement: Figure S2 — Expression of HBs proteins in the liver of transgenic mice. Western blot analysis of total protein lysates from livers of HBV transgenic mice using specific anti-HBsAg antibody. 1- female BALB/c; 2,3- female HBVTg/c; 4 – male BALB/c; 5, 6 – male HBVTg/c; 7 – female C57BL/6; 8, 9 – female HBVTg/6; 10 – male C57BL/6; 11, 12 – male HBVTg/6 mice. Equal protein loading was confirmed with anti-β-actin antibody. (TIF) [file pone.0090608.s002.tif]

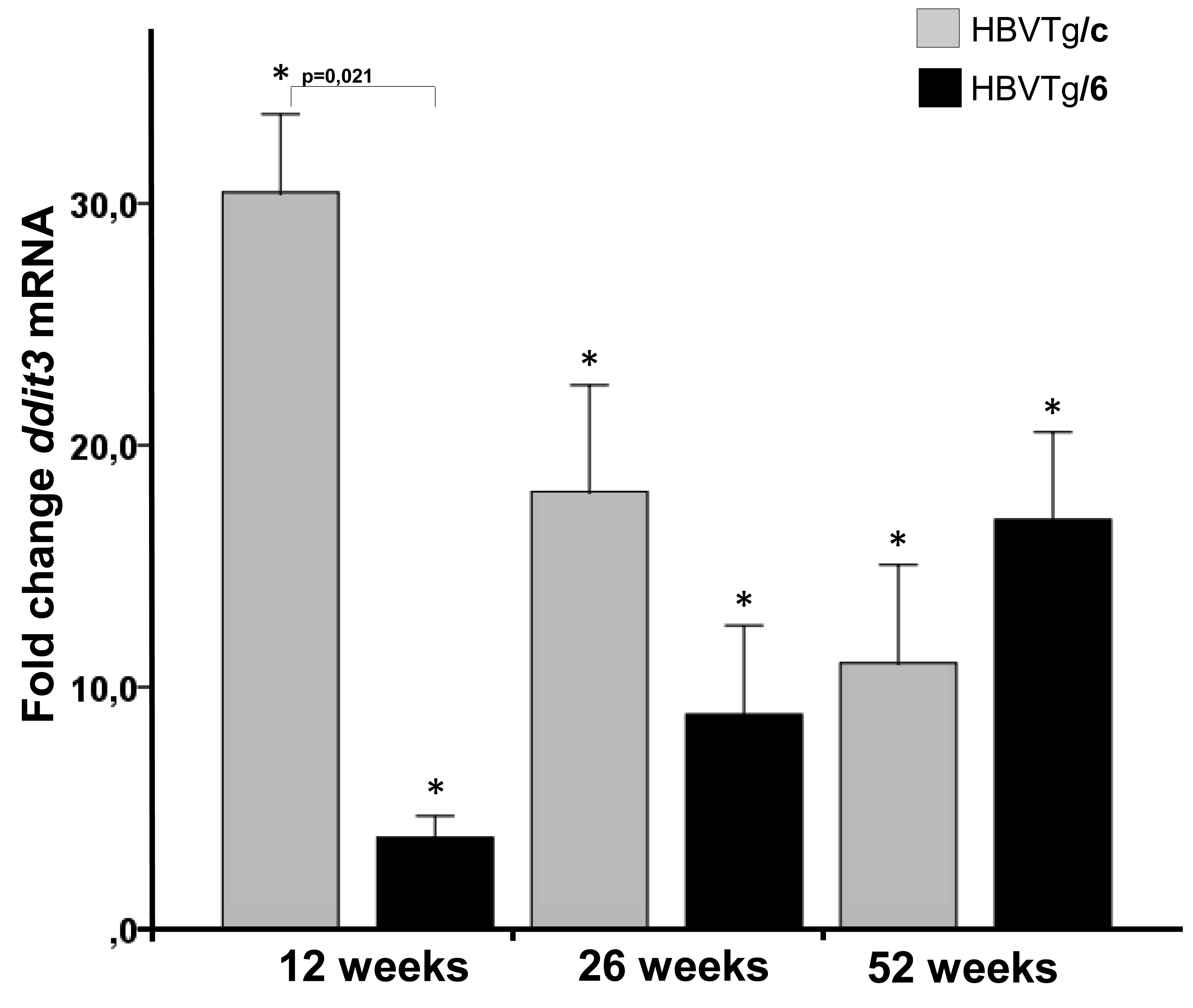

Supplement: Figure S3 — Transcriptional up-regulation of ddit3 gene in the liver of HBV transgenic mice. All data are normalized to r18S. Fold increase to wild-type animals is depicted (mean ± SEM, n = 5–10). (TIF) [file pone.0090608.s003.tif]

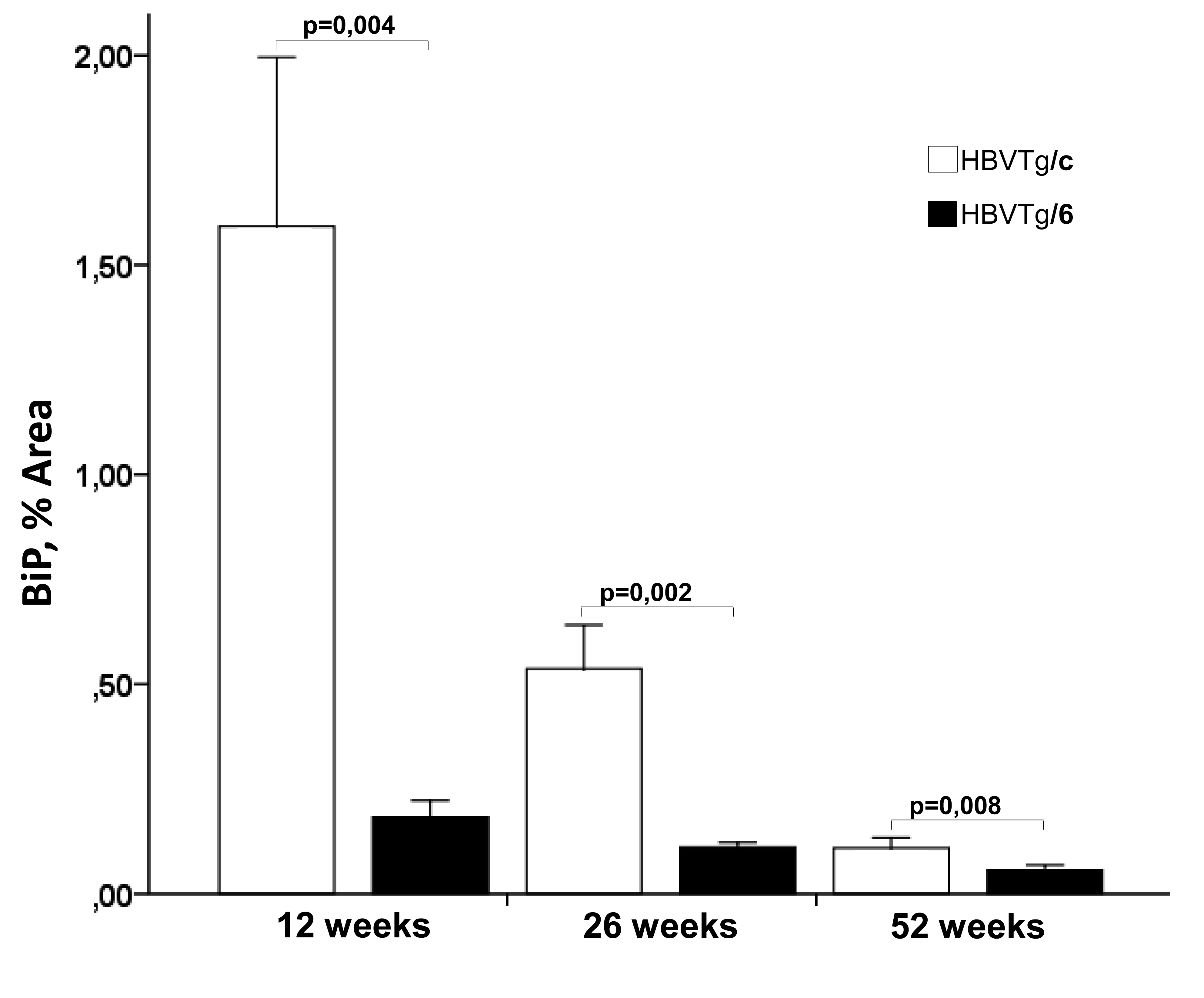

Supplement: Figure S4 — Quantification of BiP expression in the liver of HBV transgenic mice. Percentage of BiP-positive area in the whole image was estimated with ImageJ software (mean±SEM, n = 5). (TIF) [file pone.0090608.s004.tif]

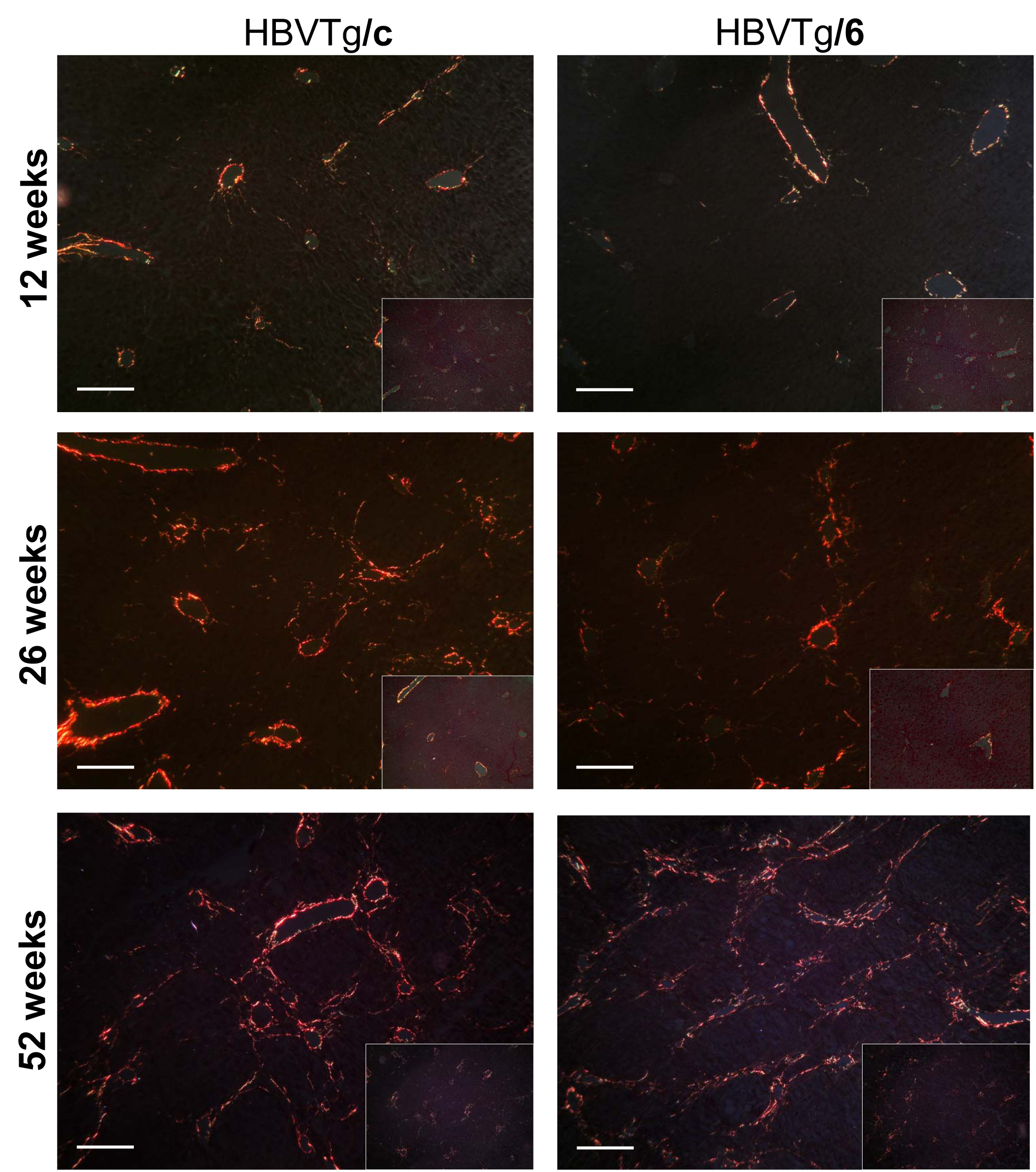

Supplement: Figure S5 — Development of fibrosis in the liver of HBV transgenic mice. Collagen fibres are detected in polarized light after Sirius red staining of 5-µm paraffin-embedded liver sections of 12-, 26-, and 52-week-old HBVTg/c and HBVTg/6 mice. Original magnification 100×, bar = 200 µm. Insets – Sirius red staining of the liver from wild-type mice. (TIF) [file pone.0090608.s005.tif]

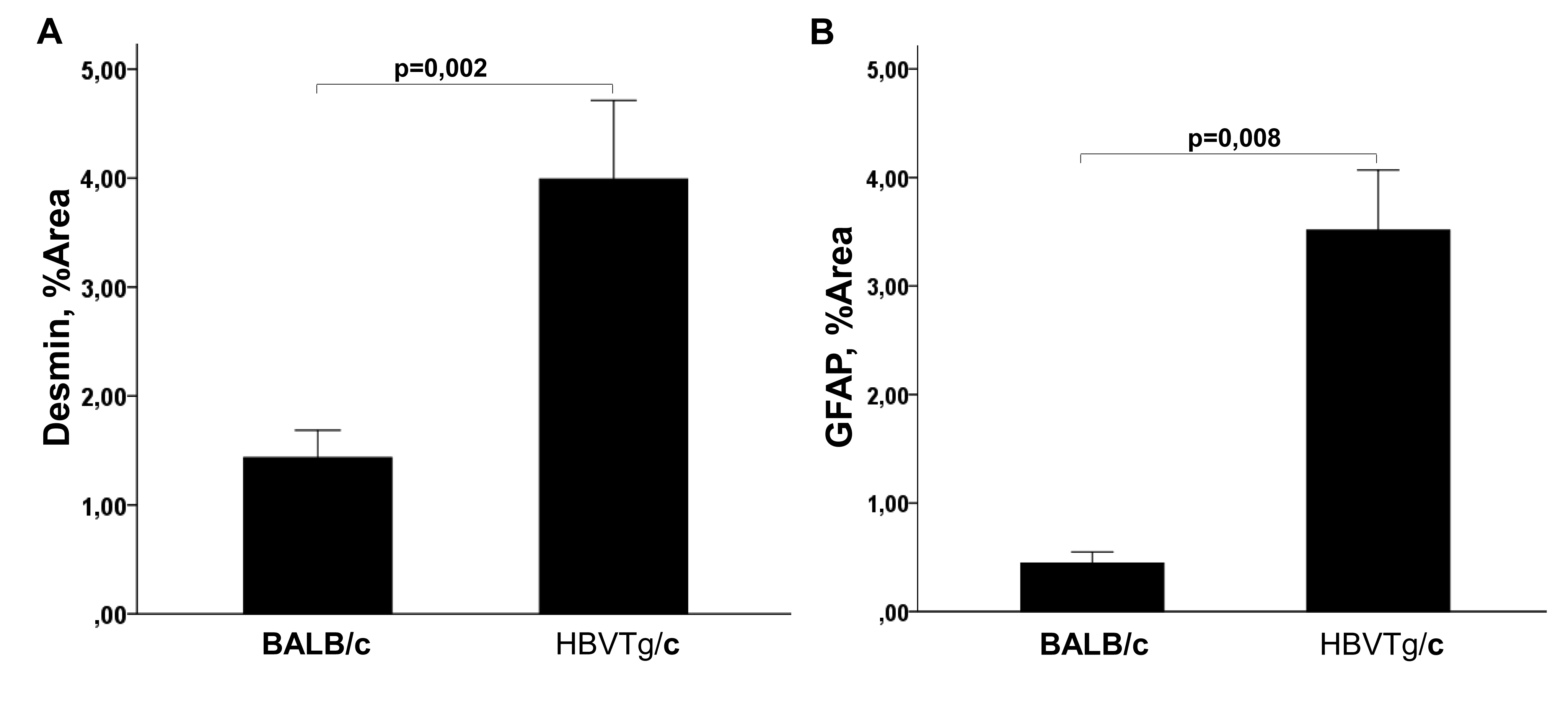

Supplement: Figure S6 — Quantification of Desmin and GFAP expression in the liver of HBV transgenic mice. Percentages of Desmin (A) and GFAP (B)-positive areas in the whole images were estimated with ImageJ software (mean±SEM, n = 5). (TIF) [file pone.0090608.s006.tif]

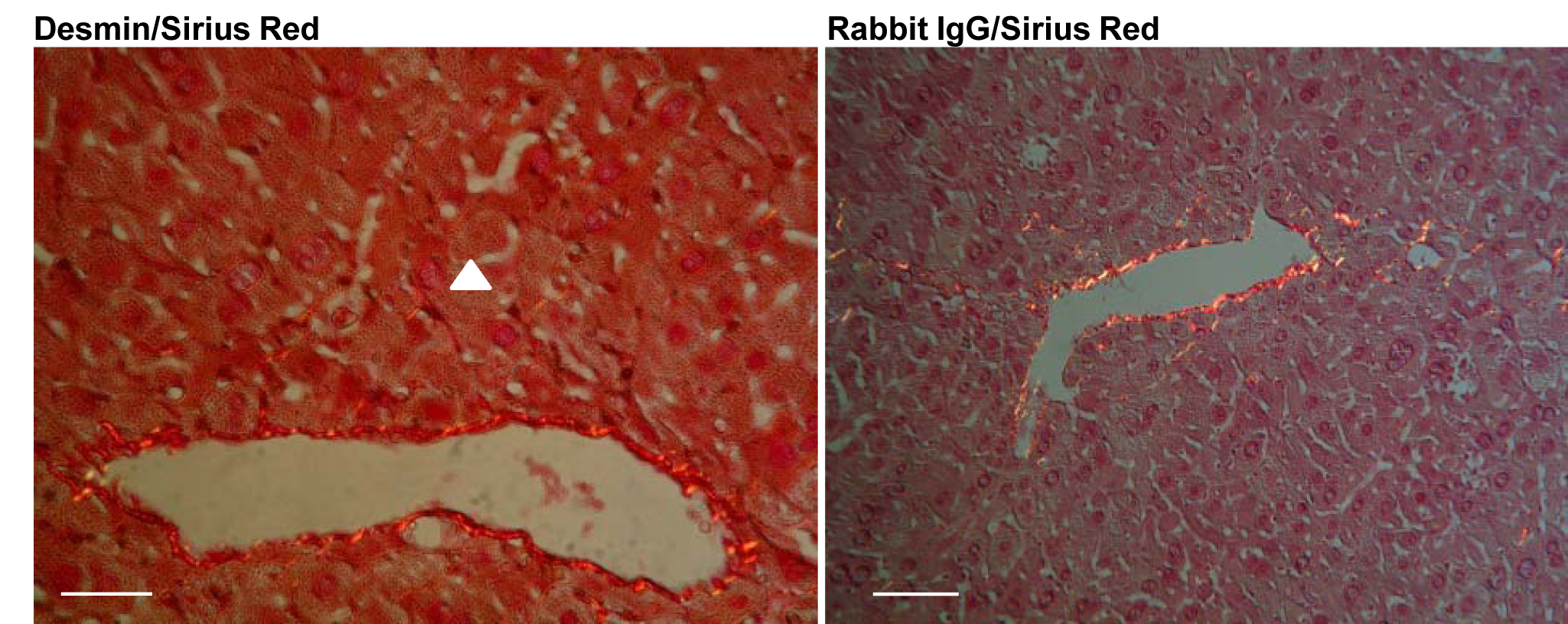

Supplement: Figure S7 — Hepatic stellate cells are major contributor to hepatic fibrosis in HBV transgenic mice. Paraffin-embedded liver section from 26-week-old HBVTg/c mouse was first stained with anti-Desmin antibody and subsequently with Sirius red. Collagen fibres are detected in polarized light, Desmin-positive staining appears in black. Original magnification 200×, bar = 100 µm. (TIF) [file pone.0090608.s007.tif]

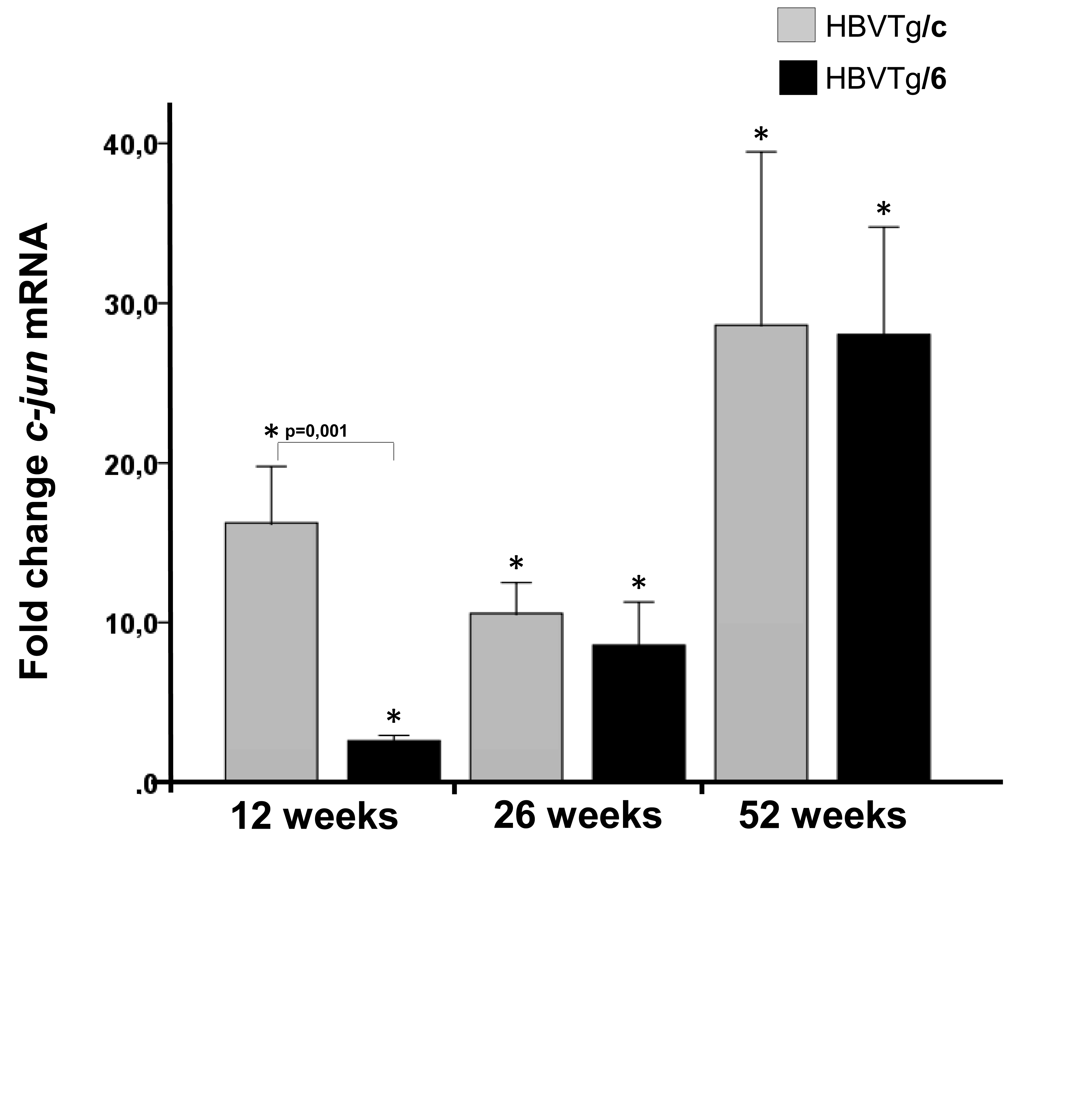

Supplement: Figure S8 — Transcriptional up-regulation of c-jun gene in the liver of HBV transgenic mice. All data are normalized to r18S. Fold increase to wild-type animals is depicted (mean ± SEM, n = 5–10). (TIF) [file pone.0090608.s008.tif]

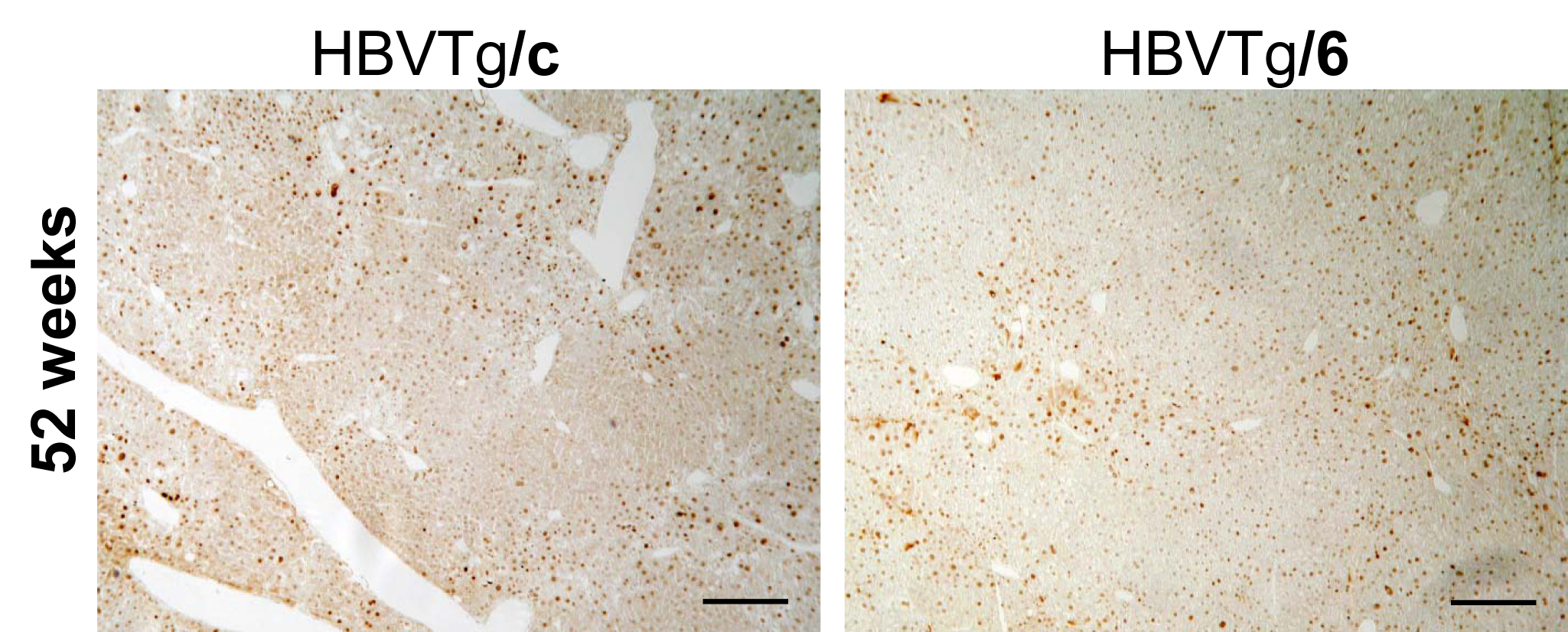

Supplement: Figure S9 — Expression of Jun protein in the liver of HBV transgenic mice. Immunohistochemical analysis of paraffin-embedded liver sections from 52-week-old mice was performed using specific anti-Jun antibody. Original magnification 100×, bar = 200 µm. (TIF) [file pone.0090608.s009.tif]
