# Supplementary figures and images for: Correction: Pathological Impact of Hepatitis B Virus Surface Proteins on the Liver Is Associated with the Host Genetic Background
Source: PLoS One. 2015 May 1;10(5):e0127375. doi: 10.1371/journal.pone.0127375 (PMC4416728; doi:10.1371/journal.pone.0127375)

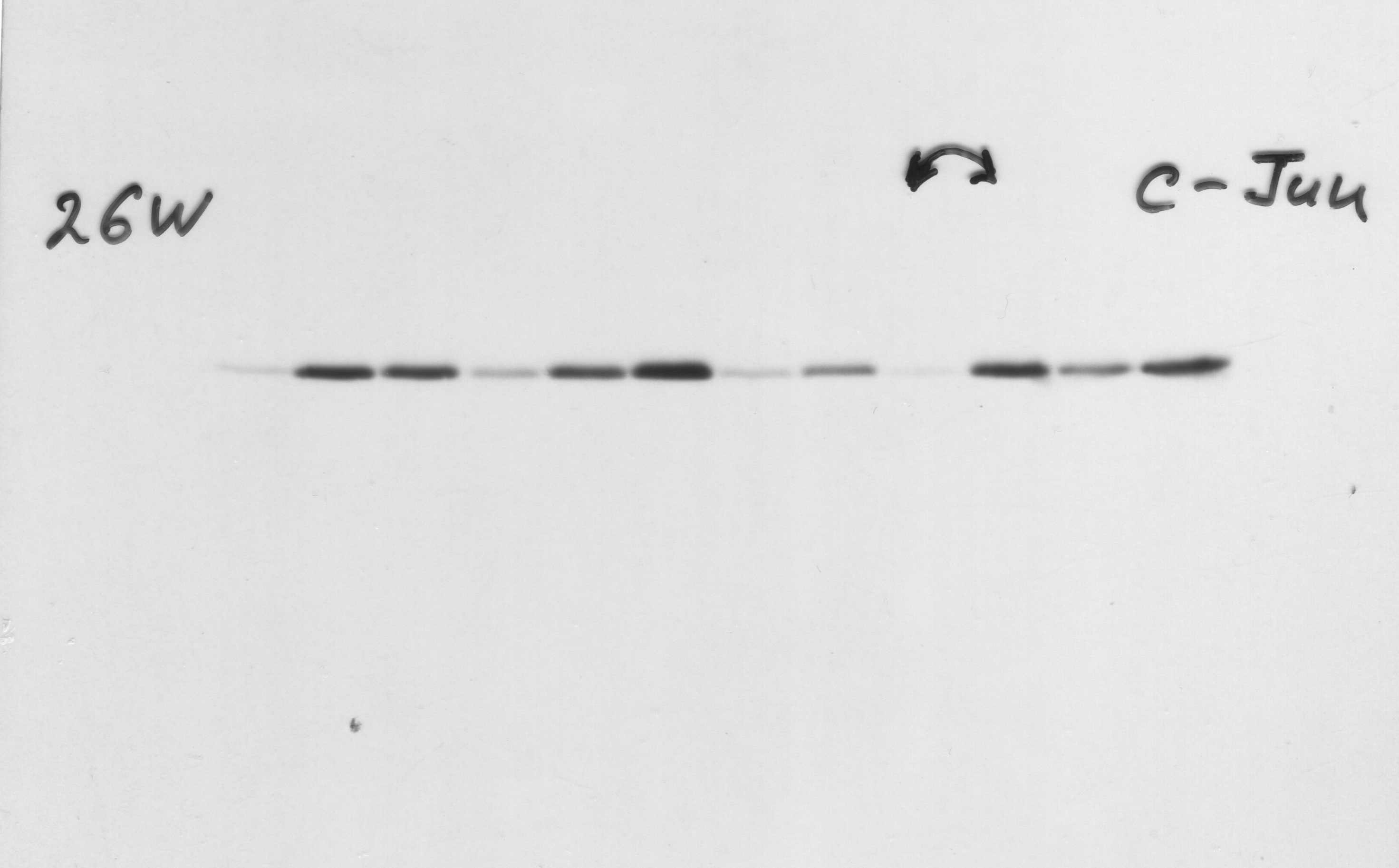

Supplement: S1 File — (TIF) [file pone.0127375.s001.tif]

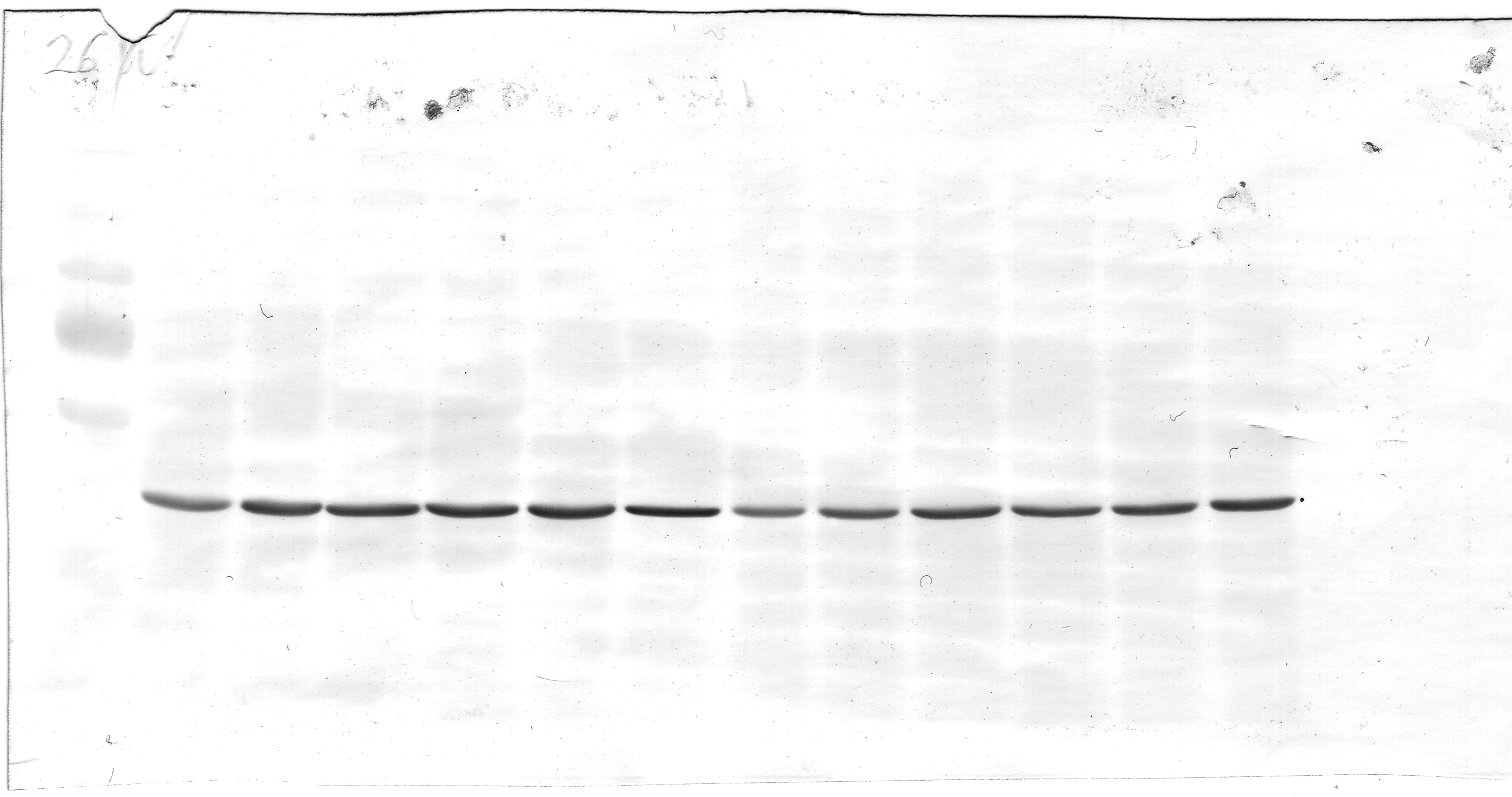

Supplement: S2 File — (TIF) [file pone.0127375.s002.tif]

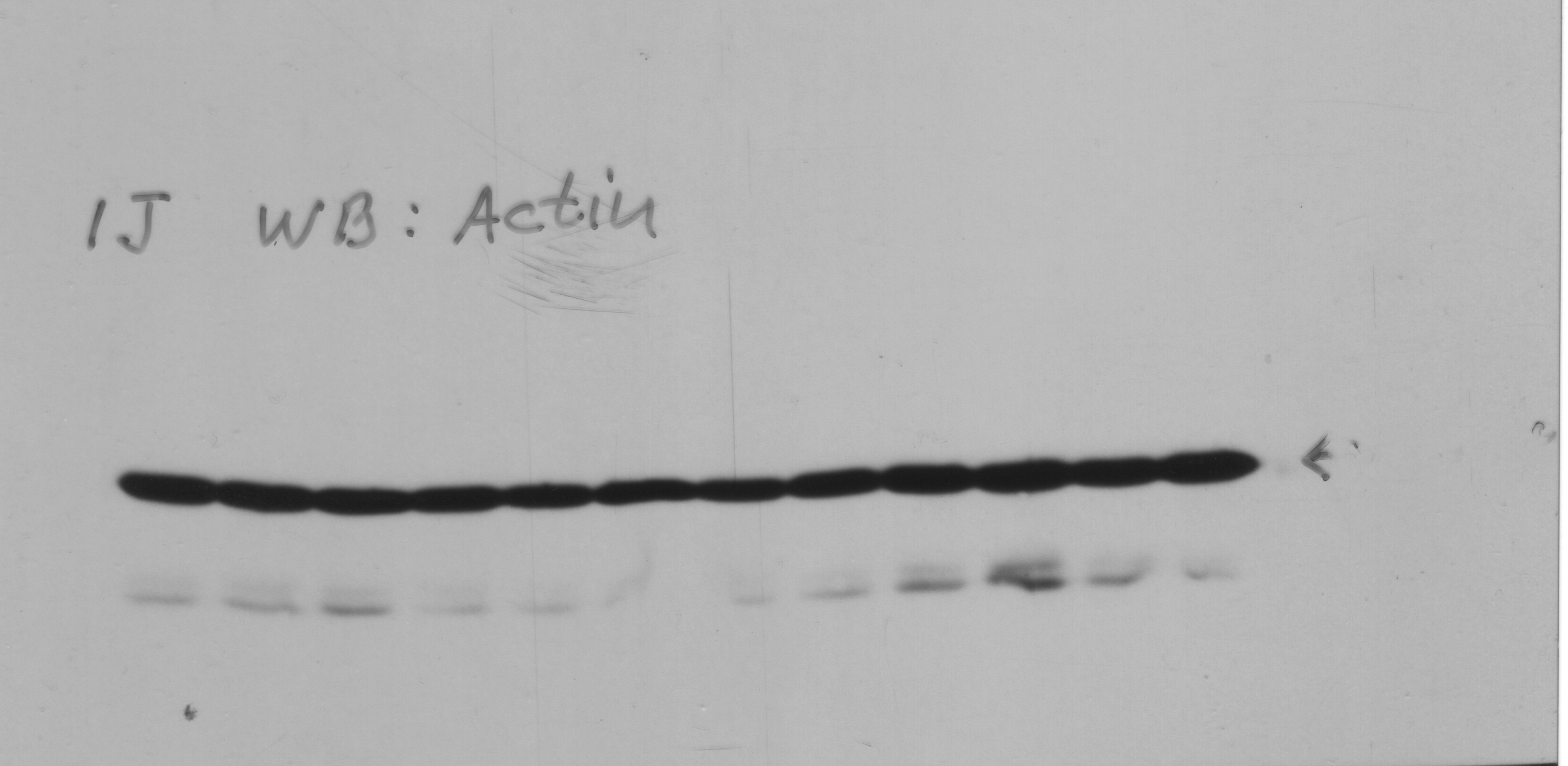

Supplement: S3 File — (TIF) [file pone.0127375.s003.tif]
